# Airway Management in Otolaryngology and Head and Neck Surgery: A Narrative Review of Current Techniques and Considerations

**DOI:** 10.3390/jcm14134717

**Published:** 2025-07-03

**Authors:** Sumrit Bola, Judith Osuji, Maria Rivero-Bosch, Rogan Corbridge

**Affiliations:** 1 Oxford University Hospital, NHS Foundation Trust, Oxford OX3 9DU, UK; 2 Royal Berkshire NHS Foundation Trust, Reading RG1 5AN, UK

**Keywords:** airway management: ENT surgery, jet ventilation, high-flow oxygen, videolaryngoscopy, tubeless field, shared airway

## Abstract

**Background**: Airway management in otolaryngology presents unique challenges due to shared airway access, altered anatomy, and specific procedural requirements. This article examines current techniques and oxygenation strategies across various ENT procedures to provide a guide for otolaryngologists. **Methods**: A narrative review was performed of the contemporary literature, focusing on airway techniques in ENT surgery, including laryngeal surgery, pediatric bronchoscopy, transoral surgery, and trauma and emergency scenarios. A systematic search for difficult airway guidelines was performed using the EMBASE, Pubmed, and Cochrane databases to examine where guidelines are published. **Results**: The key areas for specialist airway management included laryngeal surgery in the tubeless field and adjuncts for emergency situations. High-flow nasal oxygen (HFNO), jet ventilation, video laryngoscopy, and specialized tubes emerged as key technological advances, improving safety and outcomes. A systematic search identified 947 difficult airway articles across 82 publishers. These were predominantly in anesthetic journals (n = 301), with limited representation in the otolaryngology literature (n = 8) and limited guidance concerning awake surgical tracheostomies under local anesthetic. Awake tracheal intubation and emergency front-of-neck access were identified as key techniques across multiple publications. **Conclusions**: Modern ENT airway management requires multidisciplinary planning, advanced equipment familiarity, and procedure-specific techniques. Despite having the expertise to perform the gold standard, the limited otolaryngology literature on difficult airways suggests that guidelines are primarily developed by the anesthetic community.

## 1. Introduction

Airway management in otolaryngology and head and neck surgery represents one of the most challenging areas in anesthetic practice. A “shared airway” refers to a situation where the surgical team and anesthetic team are both working in the same anatomical space. There may be pathology in the airway itself (tumor or foreign body), making intubation challenging, or previous treatment, such as radiotherapy, can cause contractures that limit neck extension and access [1].

The shared airway between the surgeon and anesthetist, combined with altered anatomy from pathology or previous treatment, creates unique risks and technical challenges. The Fourth National Audit Project (NAP4) of the Royal College of Anaesthetists and the Difficult Airway Society investigated the major complications of airway management in the United Kingdom. They highlighted that over one-third of major airway complications involved head, neck, or tracheal pathology [2], and 70% of these patients presented with airway obstruction. Additionally, the increasing complexity of patient populations, including those with multiple comorbidities, necessitated more sophisticated approaches to airway management. An analysis of the Danish anesthetic database showed that emergency surgical procedures on the airway were performed 26 times more frequently in patients undergoing ENT surgeries compared to other surgeries [3], reinforcing the need for structured, clear planning in these patients.

The evolution of airway management for ENT procedures has been driven by several factors: the desire for improved surgical access, advances in minimally invasive surgery, including robotic procedures, the growing understanding of apneic oxygenation, and the development of high-flow nasal oxygen systems. Tubeless field techniques represent a paradigm shift in ENT anesthesia, whereby the endotracheal tube is removed or absent from the operative field, providing the surgeon with an unobstructed view and access to the pharynx, larynx, or upper airway [4]. This is achieved by having periods without oxygenation or with oxygen administered through a nasal prong or jet catheter.

This narrative review aims to examine the current evidence and best practices in ENT airway management across various procedures and patient populations, with a particular focus on technique selection, safety considerations, and emerging technologies that have transformed practice in recent years. As a secondary objective, the literature was also reviewed to examine where difficult airway guidelines and techniques were published.

## 2. Materials and Methods

### 2.1. Search Strategy

PubMed, Cochrane databases (Cochrane Library and Cochrane Central Register of Controlled Trials), and EMBASE databases were searched for articles, guidelines, case reports, and book chapters published between January 2015 and January 2025. The keywords for the search were “shared airway” OR “airway algorithm” OR “difficult airway” OR “difficult airway management” OR “ENT airway”.

### 2.2. Publication Screening

Having generated the search list of potentially eligible studies, duplicates were excluded and publication titles were screened for appropriateness, with inappropriate titles being excluded. The remaining papers were screened by their abstract, and data were then extracted regarding article type and journal information.

### 2.3. Selection Criteria

This review was designed in concordance with the PICOTS model to extract data. The search was to broadly identify where shared airway and difficult airway articles were published. For key ENT recommendations, the population of interest included patients with oral and airway pathology. The intervention under investigation was anesthetic and airway techniques, which were compared to standard endotracheal intubation. The primary outcomes of interest were the ability to perform surgery in the airway, taking into consideration compromises such as access and visibility. The timing encompassed studies published between 2015 and 2025, although older ENT publications were reviewed to compare documented outcomes and provide summary information and recommendations. The setting focused on planning for routine shared airway procedures and emergency algorithms.

### 2.4. Images

Generative artificial intelligence was used to create the graphic for Figure 3. The factual data points were added to Claude Sonnet 4 (Large Language Model, Anthropic 2025) to create an image of data cards for each child age.

## 3. Results

A total of 4641 airway articles were initially identified. A total of 2559 remained when the 10-year time limitation was applied, and a total of 967 were reviewed after excluding abstracts (Supplementary Figure S1). These articles were circulated across 82 publishers, the majority of which (n = 301, 31%) were published in anesthetic journals. There were a high number of publications in pediatric (n = 41, 4%) and emergency medicine journals (n = 42, 4%). There was a paucity of articles in otolaryngology journals (n = 8, 0.8%).

Across many difficult airway publications, surgical front-of-neck access was identified as the last resort after repeated failed intubation or when intubation was not possible [5,6,7,8]. Awake tracheal intubation was the gold standard where expertise was available [9], and some guidelines specified that awake surgical tracheostomy could be performed under local anesthetic [10,11].

### 3.1. Airway Selection in ENT

#### 3.1.1. Endotracheal Tubes (ETTs)

Standard adult sizing typically utilizes 7.0 mm tubes for women and 8.0 mm for men, while pediatric sizing follows established formulas of (Age/4) + 4 for uncuffed tubes and (Age/4) + 3.5 for cuffed variants [12,13]. Maintenance of the cuff pressure between 15–22 mmHg (20–30 cm H_2_O) remains critical to prevent mucosal ischemia while ensuring an adequate ventilation seal [14]. Reinforced ETTs incorporate wire-embedded construction to prevent kinking during prolonged procedures where the anesthetist has limited airway access, making them particularly valuable for extended surgeries, such as parotidectomies.

Specialized endotracheal tubes have been developed to address specific surgical challenges in ENT procedures. Nerve integrity monitor (NIM) EMG tubes incorporate bipolar stainless steel electrodes positioned on each side of the low-pressure cuffed silicone tube, enabling real-time vocal cord and recurrent laryngeal nerve monitoring during thyroid and parathyroid surgery through both audiological and visual feedback systems [15]. Microlaryngoscopy tubes (MLTs) are available in sizes ranging from 4.0 to 6.0 mm and provide improved laryngeal visualization compared to a standard ETT. The diameters are smaller, which comes at the cost of increased airway resistance, requiring higher ventilation pressures [16].

Laser-resistant tubes feature metallic foil layers that protect the underlying polyvinylchloride from laser energy, with specialized water-filled cuffs that serve dual purposes as damage indicators and fire-extinguishing systems (Table 1). Whilst they reduce the risk of airway fires, the outer diameter of these tubes is larger than a standard ETT, potentially limiting surgical access to vocal cord structures [17]. Ring–Adair–Elwyn (RAE) tubes feature preformed bends designed to position the breathing circuit away from the surgical field and are available in both north-facing (nasal) and south-facing (oral) configurations, although their predetermined insertion depth may not accommodate all patient anatomies [18].

#### 3.1.2. Jet Ventilation Systems

Jet ventilation provides high-pressure oxygen delivery through pneumatic or electronic devices, requiring total intravenous anesthesia and relying on passive expiration via chest wall recoil. This technique can be delivered through supraglottic, transglottic, or transtracheal routes depending on surgical requirements, although it presents challenges in carbon dioxide monitoring and carries risks of hypercapnia, soiling, and barotrauma [19].

Low-frequency jet ventilation (LFJV) operates at approximately 10–30 impulses per minute and is typically limited to short procedures, such as laryngoscopies or rigid bronchoscopies. Hand-triggered devices utilize rigid narrow jet nozzles that must be aligned along the airway axis to maintain effectiveness and prevent gastric distension when delivered supraglottically. During bronchoscopy procedures, the jet attaches to the side arm using 100% oxygen, though proximal positioning results in air entrainment, reducing the delivered FiO_2_ to 80–90% at the tracheal level. LFJV proves particularly valuable when clear surgical fields are required and serves as an interim measure in emergency situations using transtracheal cannulation [20].

High-frequency jet ventilation (HFJV) delivers more than 60 impulses per minute through specialized ventilators capable of producing high-frequency, low-volume breaths via small-gauge catheters. These catheters can be positioned supraglottically through suspension laryngoscopes or placed transglottically or transtracheal depending on surgical access requirements. HFJV techniques provide excellent laryngeal visualization, although FiO_2_ must be reduced to 21–30% during laser surgery to prevent airway fires [21,22]. Jet ventilation produces less airway stimulation compared to endotracheal tubes, thereby reducing sympathetic responses and laryngeal reflexes. This allows for the avoidance of neuromuscular blockade while utilizing local anesthesia as an alternative, although barotrauma remains a concern that can be mitigated by automatic pressure cutoff devices (Supplementary Table S1).

**Figure 1:**
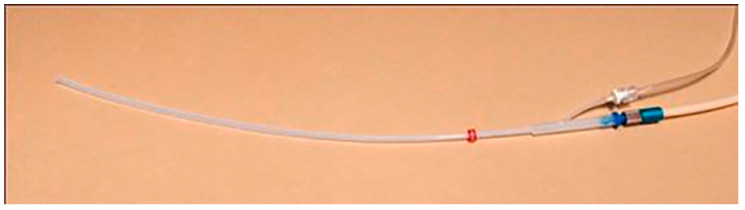
LaserJet catheter (Acutronic Medical Systems) with two lumens for delivering gas and monitoring airway pressure/ETCO2.

Supraglottic approaches provide completely tubeless surgical fields but can cause the movement of anatomical structures and require surgeons to maintain airway patency while operating. The safety features of automated HFJV, including airway pressure and end-tidal CO_2_ monitoring, are not reliably measured through this route. Transglottic ventilation minimizes vocal cord displacement but can drive blood and debris outward with the expiratory flow rather than into the tracheobronchial tree. Subglottic catheters, while mildly intrusive to the surgical field, produce less vocal cord movement and drive debris outward with the expiratory flow rather than into the tracheobronchial tree. The Laserjet (Figure 1) or Hunsaker Mon-Jet catheters come with integrated pressure monitoring capabilities and enable safe use during CO_2_ laser procedures when FiO_2_ is appropriately reduced. However, careful attention to fire prevention protocols remains essential throughout the procedure, and strategies should be in place in the case of complications (Appendix B).

#### 3.1.3. High-Flow Nasal Oxygen (HFNO)/THRIVE

**Figure 2:**
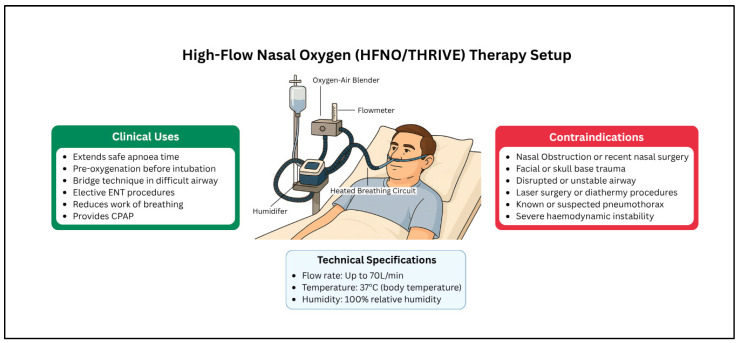
High-flow nasal oxygen equipment, uses, and contraindications.

Transnasal humidified rapid-insufflation ventilatory exchange (THRIVE) represents a significant advancement in apneic oxygenation techniques, using high-flow nasal oxygen delivery at rates of up to 70 L/min with warming and humidification capabilities [23,24,25]. This non-invasive technique (Figure 2) can extend the apneic time from the traditional 2–3 min to a mean of 14 min in patients with absent or diminished respiratory effort [26]. THRIVE achieves multiple physiological benefits, including dead space reduction and decreased work of breathing, making it particularly well-tolerated by conscious patients [27].

The clinical applications of THRIVE extend beyond simple oxygenation to continuous positive airway pressure effects that reduce atelectasis, as well as warm humidification, which limits ciliary injury. These combined effects make THRIVE particularly valuable in compromised airways, serving as a bridge technique during intubation attempts or emergency tracheostomy preparation [27]. While THRIVE does not replace established techniques such as fibreoptic intubation or awake tracheostomy, it significantly reduces patient anxiety and work of breathing before definitive airway security is achieved.

Elective ENT procedures, including laryngoscopy, can be performed using THRIVE alongside jet ventilation or as the sole oxygenation method, provided that airway patency is maintained throughout the procedure [28]. The technique proves especially valuable when combined with other airway adjuncts, such as videolaryngoscopes, enhancing both safety margins and procedural success rates [24]. Contraindications include pneumothorax, facial trauma, nasal obstruction, and uncooperative patients (Supplementary Table S1). Patient compliance is required, and it cannot be used alongside lasers or diathermy.

#### 3.1.4. Supraglottic Airways

Laryngeal mask airways (LMAs) offer significant advantages in ENT procedures through the elimination of neuromuscular blockade requirements and the provision of smooth emergence characteristics, with minimal hemodynamic response to insertion and removal [5,29]. The device can remain in situ until patients recover normal protective reflexes, allowing management in recovery areas with reduced airway irritation compared to endotracheal tubes. LMA devices prove particularly suitable for procedures involving tonsils, adenoids, nasal structures, dental work, and ear surgery, although practitioners must remain vigilant for potential complications, including laryngospasm, airway obstruction, or device displacement.

Clinical applications extend beyond routine airway management to include rescue scenarios, where LMA serves as a “Plan B” for maintaining oxygenation following failed endotracheal intubation attempts [30]. In these situations, the device provides a bridge, allowing decisions between patient awakening, procedure continuation with the supraglottic airway, intubation through the device using specialized equipment such as the LMA Fastrach^®^ (LMA Teleflex Medical) or LMA-ProSeal^TM^ (Teleflex Inc., Wayne, PA, USA) [30,31], or progression to surgical airway techniques. The versatility of supraglottic airways makes them valuable interim devices during challenging extubations, particularly following jet ventilation procedures where a smooth transition to spontaneous ventilation is desired.

Contraindications and limitations must be carefully considered, particularly in patients with aspiration risks or obesity, where higher airway pressures may be required and accidental gastric insufflation becomes more likely [32]. The risk–benefit assessment should account for procedure duration, patient positioning, and airway soiling or bleeding, with management strategies for complications (Appendix B). Second-generation supraglottic airways incorporating design features, such as higher-pressure seals and gastric tube insertion ports, provide enhanced aspiration protection compared to first-generation devices, making them more suitable for complex ENT procedures where bleeding or secretions may be encountered. A detailed airway selection table is described in Supplementary Table S1.

### 3.2. Situation-Specific Management Strategies

#### 3.2.1. Tubeless Field Techniques

For improved access to the pharynx, larynx, and upper airway, periods of apneic ventilation can be used whereby the endotracheal tube is intermittently removed and reinserted under direct vision. This apneic period causes desaturations, which can be poorly tolerated in young, obese, or frail patients. Both jet ventilation and HFNO/THRIVE represent viable oxygenation options for improved safety and extended apneic times during tubeless anesthesia, with laser and electrocautery being used at reduced FiO_2_ levels [4].

Comprehensive planning must include re-intubation and surgical airway planning if adequate oxygenation is not achieved or if the airway becomes obstructed [23]. These plans may include an initial LMA, with a surgeon to extubate under anesthesia followed by a direct laryngoscopy or progression to supraglottic jet ventilation. A rescue plan would involve keeping an ETT open for surgical intubation in the event of bleeding or poor compliance. Discussions should also address the role of laryngeal mask airways for gradual emergence in recovery and post-operative monitoring on the ward or in the ICU [23]. Local anesthetic application is essential in these scenarios to prevent laryngospasm and maintain airway patency throughout the procedure.

#### 3.2.2. Pediatric Bronchoscopy Considerations

**Figure 3:**
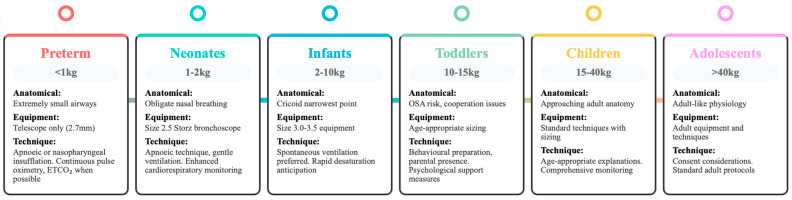
Age-specific recommendations for pediatric bronchoscopy.

Age-specific approaches to pediatric bronchoscopy reflect the significant anatomical and physiological differences that influence both equipment selection and technique application (Figure 3). Due to the size of even the smallest bronchoscopes, infants weighing less than 1 kg can only tolerate telescope-only bronchoscopy (outer diameter of 2.7 mm) [33]. These extremely small patients can be managed using either apneic techniques (intermittent extubation in a sedated patient) or the maintenance of anesthesia through nasopharyngeal insufflation of sevoflurane and oxygen while preserving spontaneous ventilation.

Premature infants weighing 1–2 kg typically require a size 2.5 Storz ventilating bronchoscope, while older children can accommodate standard Storz bronchoscopes with appropriate size selection. Foreign body removal procedures typically employ rigid bronchoscopy techniques (Table 2), though some centers have successfully utilized flexible bronchoscopy with Dormia baskets for extraction when anatomical considerations favor this approach, and fiberoptic bronchoscopy through laryngeal mask airways offers particular advantages in difficult airway scenarios or when diagnostic visualization is the primary goal [34]. The choice between techniques must consider not only the size and location of foreign materials but also the risk of pushing objects distally, making spontaneous ventilation typically preferable to positive pressure approaches.

#### 3.2.3. Bleeding Airway Management

Establishing an airway in active bleeding scenarios, such as epistaxis, bleeding malignancies, and post-tonsillectomy hemorrhage, may paradoxically result in further bleeding, aspiration, or even death [35]. Immediate priorities include hemorrhage control, the maintenance of oxygen delivery, fluid and blood product resuscitation, and the correction of any underlying coagulopathy. The presence of airway contamination reduces the rate of intubation success; therefore, adequate suction must be available (at least two large rigid suction catheters) [36]. This dual suction system enhances visualization during laryngoscopy but limits working space and may interfere with oxygen delivery depending on the direction and volume of bleeding [36].

Technical considerations for bleeding airways include the use of second-generation supraglottic airways, such as the i-gel or ProSeal LMA (Table 2). These incorporate design features to minimize aspiration risk through higher-pressure seals and dedicated ports for nasogastric tube insertion [37]. These devices may also provide some degree of tamponade for upper airway bleeding sources. Cricothyroid identification and marking should be performed early in the assessment, with clear decision-making regarding the safety of awake front-of-neck access versus rapid sequence induction and intubation, potentially utilizing suction-assisted techniques if initial approaches fail [35].

The suction-assisted laryngoscopy and decontamination (SALAD) technique has been described to facilitate endotracheal intubation and prevent aspiration in contaminated airways [38]. Blood and debris should be removed using Magill forceps under direct vision while avoiding further airway trauma or inadvertently pushing foreign material deeper into the respiratory tract.

**Table 2:** Challenges and techniques addressing different ENT scenarios.

Scenario	Primary Challenges	First-Line Techniques	Alternative Options	Emergency Backup
Laryngeal Surgery	Shared airway, tubeless field	MLT, Reinforced ETT	Jet ventilation, HFNO	Surgical tracheostomy
Pediatric Bronchoscopy	Small airways, limited reserve	Age-appropriate sizing, spontaneous ventilation	Apneic techniques, Flexible scope through LMA	Emergency intubation
Foreign Body Retrieval	Impaction risk, soiled airway	Rigid bronchoscopy, spontaneous ventilation	Flexible bronchoscopy with Dormia basket	Surgical removal
Tracheal	Lower airway access, gas trapping	Crossfield intubation, jet ventilation	HFNO with spontaneous ventilation	Emergency FONA
TORS	Limited post-docking access	Nasal reinforced ETT, full paralysis	Emergency undocking Protocols, ETT	Surgical airway
Neck Trauma	C-spine injury, soiled airway, neurological	Videolaryngoscopy with MILS	Awake intubation, iLMA, Awake FONA	Emergency FONA
Tonsillectomy	OSA patients, bleeding risk	LMA vs. cuffed ETT	Reinforced tubes	Re-intubation protocols
Bleeding Airway	Hemorrhage, aspiration risk	Second-generation SAD, RSI with suction	Awake FONA	Emergency FONA
Stridor	Time pressure, unknown anatomy	Videolaryngoscopy, HFNO, steroids, nebulizers	Awake intubation, Awake FONA	Emergency FONA

TORS: transoral robotic surgery. MLT: microlaryngoscopy tube. ETT: endotracheal tube. LMA: laryngeal mask airway. HFNO: high-flow nasal oxygen. RSI: rapid sequence induction. FONA: front-of-neck access. MILS: manual in-line stabilization.

#### 3.2.4. Trauma Airway Management

Cervical spine considerations require a careful balance between spine protection and successful airway management. Manual in-line stabilization (MILS) is performed by an assistant to maintain neutral head and neck alignment by cradling the mastoid processes and occiput [39] whilst an airway is established. Flexible laryngoscopy or videolaryngoscopy, particularly with hyperangulated devices, offers significant advantages in trauma scenarios by eliminating the need for manual alignment of the oral, pharyngeal, and laryngeal axes that traditional direct laryngoscopy requires. Recent systematic reviews demonstrate reduced overall failed intubation rates compared to direct laryngoscopy [40,41].

The intubating laryngeal mask airway (iLMA) offers several advantages for patients requiring cervical spine immobilization, providing a rapidly inserted channel that allows continued oxygenation while enabling time for fiberoptic intubation through the device [35]. The protected track for the endoscope also shields instrumentation from bleeding or contaminated areas within the upper airway. Additional techniques, such as retrograde intubation and transtracheal jet ventilation, may prove valuable in specific trauma presentations where conventional approaches are contraindicated or unsuccessful.

## 4. Discussion

Modern ENT airway management has been transformed by technological advances and evidence-based safety protocols, which have improved surgical access in oral, oropharyngeal, and airway surgery. Device selection requires the matching of techniques to procedural requirements, patient characteristics, and institutional capabilities, with an emphasis on clear communication protocols for safe outcomes.

High-flow nasal oxygen represents a paradigm shift, extending safe apneic periods from 2–3 min to 10–15 min or longer whilst reducing dependence on endotracheal intubation and jet ventilation systems. Having been used in the intensive care setting for some time, its use in surgery is still evolving, although it has become essential in maintaining safety margins across different clinical scenarios, including the emergency airway, and as an adjunct if performing an awake surgical tracheostomy.

Videolaryngoscopy, which is the use of a fiberoptic instrument or video camera laryngoscope, has become a widely accepted tool in airway management [42]. It has been described in many ENT scenarios, particularly those with anticipated difficulty, cervical spine considerations, or bleeding risks, representing a focus on enhancing visualization capabilities and improving first-pass success rates in anatomically distorted airways. Awake intubation with videolaryngoscopy and awake FONA under local anesthesia consistently emerge as the gold standard for severe airway compromise.

Although not a primary objective, a search of difficult airway publications demonstrated a wide dissemination disparity; 301 articles (31%) appeared in anesthetic journals versus only eight (0.8%) in otolaryngology publications. This reflects current difficult airway guideline development, which primarily occurs in the anesthetic community. ENT recommendations have emerged from specialized procedure-based articles reflecting subspecialty expertise with limited broader applicability, for example, laryngologists performing tubeless field surgery using approaches unsuitable or not needed for otology procedures. This is also likely to reflect the different equipment and expertise in each ENT center, making detailed guideline development difficult. Balancing surgical access against airway security drives clinical decision-making through sophisticated risk–benefit analysis, incorporating surgical demands, pathological factors, patient status, and institutional resources.

Limitations: The search strategy was for a narrative review, so article selection was subject to unconscious bias and no quantitative analysis could be performed. There was no assessment of study quality, and a range of articles, including small case series, were included, which may influence recommendations. Guidance is also related to equipment, which can vary across different ENT and surgical centers.

## Figures and Tables

**Table 1:** Airway device selection.

Device Type	Primary Indications	Key Advantages	Important Limitations	Safety Requirements
Standard ETT	Routine procedures, secure airway	Familiar, reliable, secure	May obstruct surgical field	Standard monitoring
MLT	Laryngeal surgery, limited access	Improved surgical visualization	Higher resistance, limited sizes	Adequate ventilation pressures
Laser Tubes	CO_2_ laser procedures	Laser protection, reduced fire risk	Bulky, expensive, limited access	FiO_2_ reduction, water-filled cuff
RAE Tubes	Facial/oral surgery	Circuit positioning	Fixed depth, anatomical variability	Endobronchial intubation risk
NIM Tubes	Thyroid surgery Parotid Surgery	Nerve monitoring	Equipment, Positioning critical	Electrode contact verification
Reinforced ETT	Long procedures, Position changes	Kink resistance	Higher cost, bulkier	Regular position checks
LMA (Standard)	Short procedures, difficult airway rescue	No paralysis required, smooth emergence	Aspiration risk, displacement	Appropriate selection criteria
Second-Gen SAD	Bleeding scenarios, higher pressures	Aspiration protection	Complex insertion	Proper sizing and positioning
HFNO/THRIVE	Apneic oxygenation, awake procedures	Extended apnea time, patient comfort	No pressure monitoring	Patent upper airway
Manual Jet	Emergency, limited equipment	Simple setup, immediate availability	No monitoring, pressure variability	Experienced operator essential
Automated Jet	Complex procedures, safety priority	Pressure monitoring, automated cutoffs	Complex setup, training required	Proper calibration and limits

SAD—supraglottic airway device. RAE—right angles endotracheal. ETT—endotracheal tube. HFNO—high-flow nasal oxygen.

## Supplementary Materials

The following supporting information can be downloaded at: https://www.mdpi.com/article/10.3390/jcm14134717/s1, Figure S1: Narrative search of difficult airway articles. Table S1: Summarized airway techniques.

## Abbreviations

The following abbreviations are used in this manuscript:

LFJV	Low-Frequency Jet Ventilation
HFJV	High-Frequency Jet Ventilation
HFNO	High-flow Nasal Oxygen
MILS	Manual in-line Stabilization
THRIVE	Transnasal Humidified Rapid-Insufflation Ventilatory Exchange
FONA	Front-of-Neck Access

## Appendix A

**Table A1:** Summary of key conclusions.

Key Finding	Clinical Implication
High-flow nasal oxygen has become foundational technology	Extends safe apneic periods and reduces reliance on complex ventilation techniques whilst maintaining excellent safety margins across diverse clinical scenarios
Videolaryngoscopy should be considered first-line	Most appropriate for ENT procedures, particularly those with anticipated difficulty, cervical spine considerations, or bleeding risks
Automated jet ventilation systems have significantly improved safety	Compared to manual techniques, but application requires appropriate training and institutional protocols to minimize complications
Multidisciplinary team protocols are needed	Clear communication protocols are essential for safe outcomes in this high-risk specialty area, as evidenced by a higher rate of complications for patients undergoing ENT procedures
Pediatric procedures require specialized age-specific approaches	Must account for anatomical, physiological, and psychological differences from adult patients, with particular attention to equipment sizing and monitoring requirements
Emergency presentations demand flexible, well-rehearsed strategies	Requires multiple backup options and immediate surgical availability, emphasizing preparation over improvisation
Publication patterns suggest evolving clinical practice	Increasing reliance on advanced medical airway management techniques and decreasing reliance on surgical airways reflect improved airway adjuncts and advanced airway techniques
Technology integration continues to evolve	Optimal combinations of HFNO, videolaryngoscopy, and automated monitoring systems show promise for further safety improvements
Quality improvement	The standardization of protocols, training methods, and outcome measurement represents a critical area for continued development and research

## Appendix B

**Table A2:** Complication management and prevention strategies.

Complication	Risk Factors	Prevention Strategies	Immediate Management	Long-Term Considerations
Pneumothorax	High pressures, jet ventilation	Pressure monitoring, gradual increases	Chest drain, ventilation adjustment	Follow-up imaging, respiratory function
Gas Trapping	Narrow airways, inadequate expiration	Sufficient expiratory time, low pressures	Ventilation cessation, manual evacuation	Airway assessment, technique modification
Hypoxemia	Equipment failure, shared airway	Backup oxygenation, continuous monitoring	Immediate ventilation, consider FONA	Root cause analysis, protocol review
Airway Fire	Laser surgery, high FiO_2_	FiO_2_ limitation, fire protocols	Laser cessation, debris removal	Airway assessment, multidisciplinary team
Laryngospasm	Inadequate anesthesia, irritation	Proper depth, topical anesthesia	Positive pressure, succinylcholine	Extubation planning, follow-up
Aspiration	Bleeding, inadequate protection	Appropriate device selection	Suction, positioning, intubation	Respiratory support, antibiotics
Barotrauma	Excessive pressures, prolonged exposure	Automated systems, pressure limits	Pressure reduction, supportive care	Imaging studies, respiratory follow-up
Equipment Failure	Complex systems, user error	Regular maintenance, training	Alternative techniques, backup plans	Equipment review, staff education
