# Supplementary material for: Airway Management in Otolaryngology and Head and Neck Surgery: A Narrative Review of Current Techniques and Considerations
Source: J Clin Med. 2025 Jul 3;14(13):4717. doi: 10.3390/jcm14134717 (PMC12250090; doi:10.3390/jcm14134717)

Supplementary material

Supplementary Figure S1: Publication search for 2015-2025 for articles with "shared airway" OR "airway algorithm" OR "difficult airway" OR "difficult airway management" OR “ENT airway”

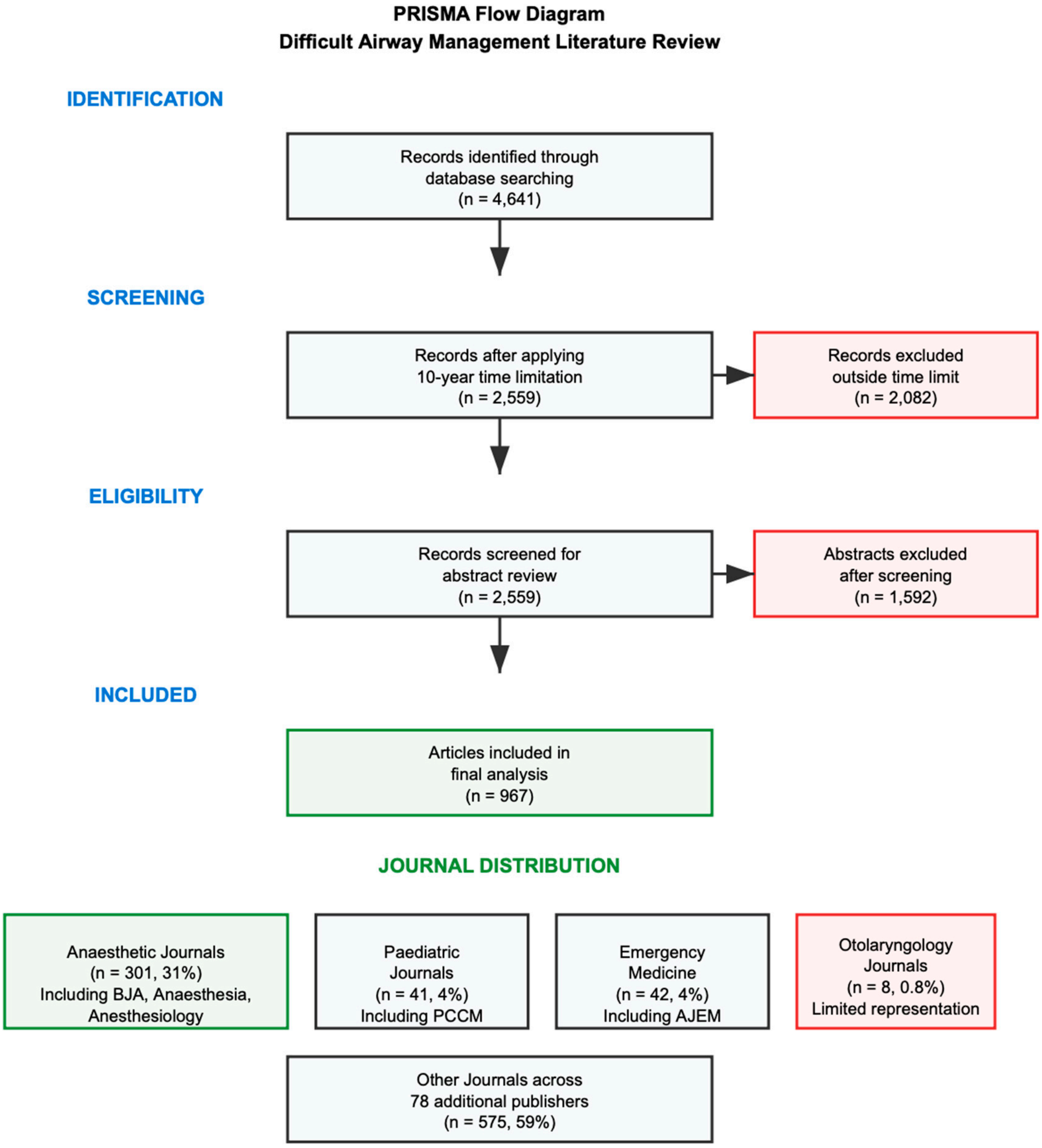

Supplement: Supplementary file 1 [file jcm-14-04717-s001.zip › jcm-3709581-supplementary.pdf]
