# Supplementary material for: Airway Management in Otolaryngology and Head and Neck Surgery: A Narrative Review of Current Techniques and Considerations
Source: J Clin Med. 2025 Jul 3;14(13):4717. doi: 10.3390/jcm14134717 (PMC12250090; doi:10.3390/jcm14134717)
Supplement: Supplementary file 1 [file jcm-14-04717-s001.zip › Table S1 -Airway techniques summarised.pdf]

Airway	Description	Examples	Pros	Cons	Procedures	Laser safe?
Transnasal Humidified Rapid-Insufflation Ventilatory Exchange (THRIVE)	High flow oxygen delivered at rates 10-70L/min via specialised nasal cannulae.<br><br>Apnoeic ventilation<br><br>Peri-procedural oxygenation-can buy time before securing airway	Optiflow™<br><br>Vapotherm®<br><br>Airvo™	Airway humidification<br><br>Unobstructed surgical view<br><br>Fixed performance permitting accurate delivery of 100% 0_2<br><br>Improved safety of conscious sedation<br><br>Decreases work of breathing	Unprotected airway<br><br>Contradicted in complete airway obstruction and very obese patients.<br><br>Nasal irritation<br><br>Patient compliance required	Laryngeal tumour resection<br><br>Oesophageal dilatation<br><br>Microlaryngoscopy / Panendoscopy<br><br>Foreign Body	No
Supraglottic airway	Device used to maintain upper airway patency and enable ventilation, sits just above the glottic opening	LMA<br><br>Flexible-LMA<br><br>i-gel®	Avoids the complications of intubation<br><br>Less coughing on emergence/waking<br><br>Neuromuscular blocking agents not required	Unprotected airway / risk of aspiration<br><br>Easily displaced<br><br>Obstructs surgical view<br><br>Risk of laryngospasm	Tonsillectomy<br><br>Adenoidectomy<br><br>Middle ear surgery	No
Endotracheal tube (ETT)	Device used to secure the airway allowing controlled or occasionally spontaneous ventilation<br><br>These can be orally or nasally inserted	South/North RAE<br><br>Nerve integrity monitor tubes (NIM)<br><br>Reinforced<br><br>Laser ET tube	Protected airway<br><br>Shaped for procedure<br><br>Monitoring recurrent laryngeal nerve<br><br>Resistant to kinking	Surgical view obstruction<br><br>Laser tube used with FiO_2<30%	Parotidectomy<br><br>Submandibular gland excision<br><br>Thyroidectomy<br><br>Microlaryngoscopy	Only laser ETT
	4-6mm endotracheal tube with a longer length than normal	Microlaryngoscopy tube	Improved surgical view compared to standard ETT<br><br>Protected airway<br><br>Standard anaesthetic techniques and breathing system can be used	Reduced access to posterior 1/3 glottis<br><br>High resistance	Microlaryngoscopy<br><br>Oesophagoscopy<br><br>Laryngoscopy	No
High frequency Jet Ventilation (HFJV)	Delivery of small tidal volumes (1-3mls/kg) from a high pressure jet at supraphysiological frequencies (1-10Hz) followed by passive expiration (therefore requires a patent upper airway)<br><br><u>General risks with all types of HFJV:</u><br><br>Barotrauma<br><br>Exposure to dry gas (mucosal trauma, atelectasis)<br><br>Hypercapnia<br><br>Potential airway soiling (debris/secretions)	<u>Supraglottic:</u><br>Cannula attached to surgical laryngoscope	Completely unobstructed view<br><br>No extraneous flammable material in airway	Unprotected airway<br><br>Ventilation dependant on alignment from surgeon<br><br>Vocal cord movement<br><br>Unable to monitor airway pressures or end tidal CO2<br><br>Air entrainment due to proximity of jet leads to variable Fi02	Pharyngoscopy<br><br>Laryngoscopy	Yes<br>FiO_2<br>21%
		<u>Transtracheal:</u><br>Use of specialised cannula inserted percutaneously	Minimal entrainment of air therefore good control of Fi02<br><br>Unobstructed surgical view	Problematic with small diameter stenosis. Cannula may kink<br><br>Unable to monitor airway pressures or end tidal CO2	Short procedure in laryngectomy patient	
		<u>Subglottic/ Transglottic:</u><br>Use of specialised catheter placed at laryngoscopy by anaesthetist	Minimal entrainment<br><br>CO2 and airway pressure monitoring possible<br><br>Good surgical access<br><br>Less vocal cord movement	May obstruct surgical view	Microlaryngoscopy	
Low frequency jet ventilation (LFJV)	Use of rigid bronchoscope or laryngoscope to ventilate by means of a pressure regulator and hand-held on/off valve<br><br>As above can be supra/trans or subglottic	Sanders<br><br>Manujet	Simple gas injector- manual or Jet<br><br>Side-arm in some models allows use of anaesthetic vapours	Mobile operative field<br><br>Barotrauma<br><br>Air entrainment<br><br>Requires patent upper airway for passive expiration	Rigid Bronchoscopy<br><br>Emergency Cricothyroidotomy	Yes
